# Possible SARS‑CoV‑2 and Mycoplasma pneumoniae co‑detection during the early COVID‑19 pandemic: diagnostic challenges in a resource‑limited setting

**DOI:** 10.1099/acmi.0.001285.v3

**Published:** 2026-11-11

**Authors:** Hidehiro Watanabe, Takehito Kobayashi, Yasuyuki Takebayashi, Hideaki Takahashi, Hiroaki Fujita, Yusuke Watanabe, Itaru Nakamura

**Affiliations:** 1 Department of Infection Control and Prevention/Department of Infectious Diseases, Tokyo Medical University Hospital, Tokyo, Japan; 2 Department of Infection Control and Prevention/Department of Infectious Diseases, Tokyo Medical University Ibaraki Medical Center, Ibaraki, Japan

**Keywords:** diagnostic reasoning, early COVID-19 pandemic, *Mycoplasma pneumoniae*, possible co-detection, SARS-CoV-2

## Abstract

During the early Coronavirus disease 2019 (COVID-19) pandemic, diagnostic pathways were disrupted by limited access to severe acute respiratory syndrome coronavirus 2 (SARS-CoV-2) testing, evolving clinical knowledge and substantial diagnostic uncertainty. We report a case in which *Mycoplasma pneumoniae* (*M. pneumoniae*) and SARS-CoV-2 were both detected early in the clinical course of a previously healthy 35-year-old man in April 2020. The patient met all six Japanese clinical criteria for atypical pneumonia, and chest computed tomography demonstrated multifocal ground-glass opacities compatible with atypical pneumonia. Clarithromycin was initiated on Day 4. An *M. pneumoniae* particle agglutination titre of 1 : 640 was recognized on Day 6, supporting possible *M. pneumoniae* infection, although serology alone cannot confirm acute disease. Later that afternoon, a positive SARS-CoV-2 PCR result was reported, prompting reframing of the working diagnosis towards COVID-19. Antimicrobial therapy was temporarily discontinued and subsequently adjusted. This case illustrates how early-pandemic diagnostic constraints, delayed availability of microbiological results and overlapping radiological findings created diagnostic complexity. Although cognitive biases such as anchoring may have contributed to the diagnostic shift, available information is insufficient to establish them as definitive causes. Instead, this case highlights the challenges of interpreting limited diagnostic data in a resource-restricted setting. As contemporary COVID-19 disease severity has attenuated and atypical bacterial infections have resurged, maintaining awareness of possible co-detection remains important for accurate clinical assessment.

## Data Summary

All data associated with this work are reported within the article. No additional datasets were generated.

## Introduction

During the early Coronavirus disease 2019 (COVID-19) pandemic, clinicians faced substantial diagnostic uncertainty due to limited access to severe acute respiratory syndrome coronavirus 2 (SARS-CoV-2 testing, evolving epidemiological information and constrained microbiological resources. Diagnostic pathways for respiratory infections were frequently disrupted, and clinicians often had to make decisions based on incomplete or delayed information. These circumstances created an environment in which diagnostic complexity was common, and cognitive biases may have influenced clinical reasoning, although such influences are difficult to confirm retrospectively.

*Mycoplasma pneumoniae* (*M. pneumoniae*) pneumonia is a well-recognized cause of community-acquired pneumonia in younger adults in Japan. Clinicians commonly use a six-item clinical scoring system to differentiate atypical pneumonia from typical bacterial pneumonia, and suspicion of atypical pneumonia increases when four or more criteria are met, as validated in multiple Japanese Respiratory Society (JRS) studies [1, 2]. Radiological findings of multifocal ground-glass opacities may be compatible with *M. pneumoniae* pneumonia, but these features overlap substantially with early COVID-19, particularly during the initial pandemic surge.

Reports of SARS-CoV-2 and *M. pneumoniae* co-detection were limited in early 2020, partly due to restricted access to pathogen-specific testing and partly because COVID-19 was prioritized as the dominant diagnosis. In this context, distinguishing true co-infection from sequential or incidental detection was challenging.

We present a case from April 2020 in which *M. pneumoniae* serology and SARS-CoV-2 PCR were both positive early in the clinical course. Rather than demonstrating diagnostic error, this case illustrates the diagnostic challenges inherent to early-pandemic practice, including delayed test availability, overlapping imaging findings and evolving clinical frameworks. The case also highlights considerations relevant to contemporary practice, in which atypical bacterial infections have resurged and COVID-19 severity has attenuated.

## Case presentation

### Patient information

A previously healthy 35-year-old man developed a fever on 18 April 2020 (Day 0). He had no significant past medical history and no recent travel. Fever and upper respiratory symptoms persisted through Days 1–3.

### Day 4 (22 April): outpatient visit

The patient presented with fever, cough and fatigue. Vital signs were stable, and no abnormal lung sounds were detected. Laboratory tests demonstrated leucocytopenia (WBC 3,100 per microlitre).

In Japan, clinicians frequently use a six-item clinical scoring system to differentiate atypical pneumonia from typical bacterial pneumonia [1, 2]. Suspicion of atypical pneumonia increases when four or more criteria are met. This patient met all six criteria:

1. age <60 years,
2. absence of comorbidities,
3. persistent dry cough,
4. no abnormal lung sounds,
5. lack of sputum,
6. leucocytopenia (<10,000 per microlitre).

Based on these findings, clinicians judged *M. pneumoniae* pneumonia to be the most likely diagnosis.

### Imaging

Chest X-ray was normal, and chest computed tomography revealed multiple small bilateral ground-glass opacities compatible with atypical pneumonia (Fig. 1) .

**Fig. 1.**
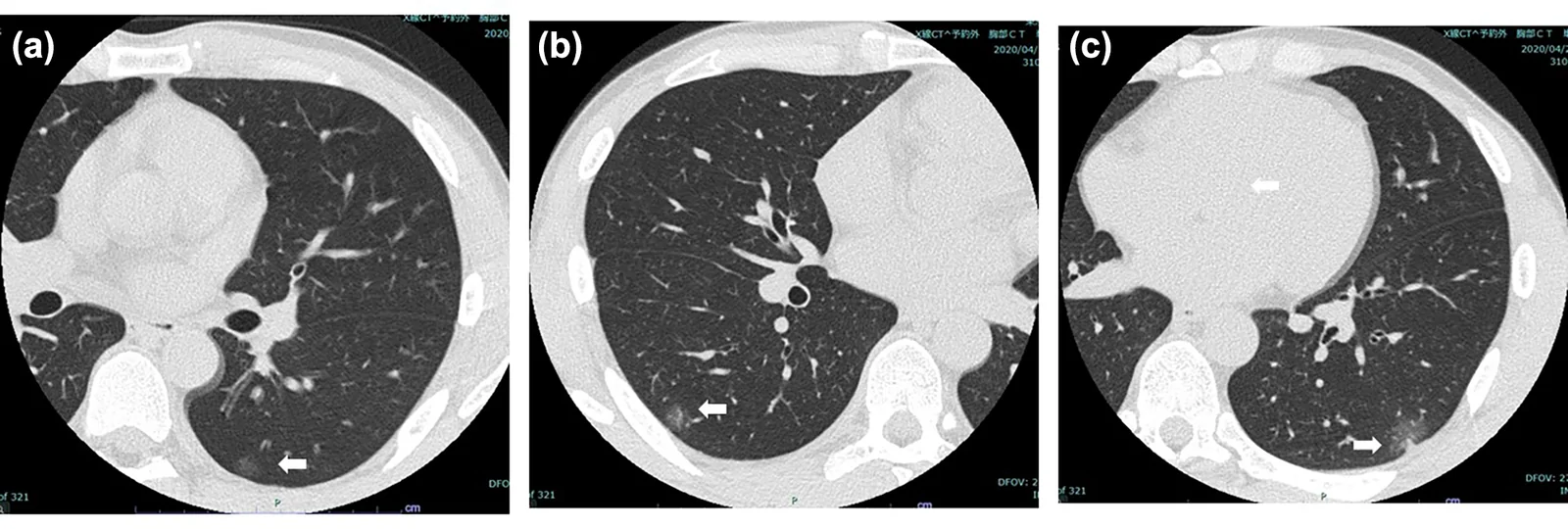
Chest computed tomography on Day 4. Axial images (a–c) show multiple bilateral 1–1.5 cm ground-glass opacities (white arrows), findings compatible with atypical pneumonia. These findings are not specific for *M. pneumoniae* or COVID-19.

The outpatient clinician interpreted these findings as consistent with *M. pneumoniae* pneumonia. In contrast, the radiologist judged the findings as not typical for COVID-19 but insufficient to rule it out.

### Initial management

Clarithromycin (CAM) was initiated, and the patient was instructed to remain at home for rest, as his clinical condition was mild (CURB-65 score: 0) and did not meet criteria for hospitalization at that time.

### Diagnostic tests submitted

SARS-CoV-2 PCR, *M. pneumoniae* particle agglutination (PA), *Chlamydophila pneumoniae* IgM and *Bordetella pertussis* IgM were submitted. Due to early-pandemic delays, results were not immediately available.

### Diagnostic assessment

On Day 4 (22 April), clinicians suspected *M. pneumoniae* pneumonia based on clinical criteria and imaging findings compatible with atypical pneumonia. At that time, *M. pneumoniae* PCR was not available at the institution, and multiplex respiratory PCR had not yet been introduced. Therefore, serology was the only available diagnostic modality.

On Day 5 (23 April) in the evening, the *M. pneumoniae* PA titre (1 : 640) was uploaded to the electronic medical record, but the result was not yet recognized by the clinical team.

On Day 6 (24 April), early in the morning, the outpatient clinician reviewed the PA result and judged that *M. pneumoniae* infection was serologically supported. Although a single PA titre cannot confirm acute infection, clinicians considered antimicrobial therapy appropriate and continued CAM.

Later that afternoon, a positive SARS-CoV-2 PCR result was reported. The working diagnosis was reframed towards COVID-19, reflecting early-pandemic diagnostic priorities and institutional requirements for isolation hospitalization. *M. pneumoniae* was reconsidered as a possible co-pathogen.

### Diagnosis and treatment

Day 6 (24 April)

Morning: *M. pneumoniae* PA titre (1 : 640) recognized; CAM continued.

15 : 00: Public health authorities notified the clinical team of a positive SARS-CoV-2 PCR result. COVID-19 diagnosis was confirmed. (At that time in Japan, all PCR-positive patients required isolation hospitalization.) Responsibility for care formally transferred to the inpatient COVID-19 team, as mandated by early-pandemic regulations. Clinicians discussed whether admission should occur the same day or the following day. Same-day admission was decided.

17 : 30: Patient contacted regarding urgent admission.

18 : 30: Hospital admission and care handover: Care was transferred from the outpatient physician to the inpatient team. Based on the initial handover emphasizing the confirmed SARS-CoV-2 PCR result, the inpatient team prioritized COVID-19 isolation and management in accordance with institutional protocols. CAM was temporarily discontinued. Intravenous fluids were initiated. At this initial juncture, clinical focus naturally centred on the urgent viral diagnosis.

19 : 30: Favipiravir was initiated according to institutional protocol.

20 : 30: Inpatient team re-evaluation occurred shortly thereafter: Later that evening, after initial admission management was established, the inpatient team conducted a comprehensive review of the patient’s electronic medical record. The team re-evaluated the outpatient clinical course, including the JRS atypical pneumonia criteria (6/6) and the elevated *M. pneumoniae* PA titre (1 : 640) recognized that morning. Recognizing that possible *M. pneumoniae* co-detection could not be ruled out, the team decided to initiate intravenous levofloxacin (LVFX) to ensure empirical coverage for potential atypical bacterial co-pathogens.

### Clinical course

The patient gradually improved over Days 7–14. A first SARS-CoV-2 PCR-negative result was obtained on Day 23 (11 May), and a second consecutive negative result on Day 25 (13 May), fulfilling the discharge criteria at that time. A detailed clinical timeline, including the diagnostic shift from suspected *M. pneumoniae* pneumonia to COVID-19, is summarized in Table 1.

**Table 1.** Timeline of clinical course, diagnostic testing and the diagnostic shift between suspected *M. pneumoniae* pneumonia and COVID-19

Day	Date	Time	Clinical events, diagnostic tests and therapeutic actions
**Day 0**	18 Apr	—	Onset of fever (37.5 °C).
**Days 1–3**	19–21 Apr	—	Persistent fever and upper respiratory symptoms.
**Day 4**	22 Apr	—	**Outpatient visit:** Physical exam normal; WBC 3,100 per microlitre. Patient met all 6 JRS atypical pneumonia criteria. **Imaging:** Chest X-ray normal; CT showed multifocal ground-glass opacities compatible with atypical pneumonia. **Treatment:** CAM was initiated; patient remained at home (mild condition). **Tests submitted:** SARS-CoV-2 PCR, *M. pneumoniae* PA, *C. pneumoniae* IgM and *B. pertussis* IgM.
**Day 5**	23 Apr	Evening	*M. pneumoniae* PA titre (1 : 640) uploaded to EMR, but not yet recognized by clinicians.
**Day 6**	24 Apr	Morning	**PA result reviewed**; *M. pneumoniae* infection considered serologically suggestive. CAM continued.
		**15 : 00**	**Public health authorities notified team of positive SARS-CoV-2 PCR; COVID-19 confirmed** (isolation hospitalization required).
		17 : 30	Patient contacted regarding admission.
		18 : 30	**Hospital admission and care handover:** Care transferred to inpatient team; COVID-19 isolation prioritized; CAM temporarily discontinued; IV fluids were initiated.
		19 : 30	Favipiravir was initiated according to institutional protocol.
		20 : 30	**Team discussion:** EMR re-evaluated (JRS criteria 6/6 and PA titre 1 : 640); LVFX was initiated due to possible *M. pneumoniae* co-detection.
**Days 7–14**	25 Apr–2 May	—	Gradual clinical improvement with resolution of fever.
**Day 23**	11 May	—	First negative SARS-CoV-2 PCR obtained.
**Day 25**	13 May	—	Second consecutive negative SARS-CoV-2 PCR obtained (fulfilled discharge criteria); patient discharged.

EMR, electronic medical record.

## Discussion

This case illustrates the diagnostic challenges encountered during the early COVID-19 pandemic, when clinicians were required to make decisions based on incomplete information, delayed test availability and evolving epidemiological understanding. In April 2020, SARS-CoV-2 PCR testing capacity in Japan was limited, turnaround times were prolonged and pathogen-specific diagnostics for atypical bacteria – such as *M. pneumoniae* PCR or multiplex respiratory PCR – were not yet available at our institution. Although external laboratories could perform these tests, outsourcing was not routinely feasible due to limited availability, prolonged turnaround times and early-pandemic operational constraints. During this period, commercial reference laboratories were prioritizing SARS-CoV-2 PCR capacity, further limiting access to other molecular diagnostics. As a result, clinicians relied heavily on clinical criteria and serology when evaluating patients with suspected atypical pneumonia.

On Day 4, the patient met all six JRS clinical criteria for atypical pneumonia, and chest CT demonstrated multifocal ground-glass opacities compatible with this diagnosis. However, interpretation of these imaging findings differed between clinicians: the outpatient physician considered the pattern consistent with *M. pneumoniae* pneumonia, whereas the radiologist judged COVID-19 possible despite atypical features. These differing assessments contributed to early diagnostic uncertainty, reflecting the substantial radiological overlap between atypical bacterial pneumonia and early COVID-19 during the initial pandemic surge. In addition, the JRS criteria have been validated in multiple studies and show high diagnostic accuracy when four or more parameters are met [1, 2]. Because the patient satisfied all six criteria, the pre-test probability of atypical pneumonia was considered high, supporting the initial administration of CAM at a time when molecular diagnostics were unavailable. The PA titre of 1 : 640 recognized on Day 6 further suggested possible *M. pneumoniae* infection, although a single serological measurement cannot confirm acute disease. The absence of molecular testing represents an important limitation of early-pandemic diagnostic practice.

Later that afternoon, a positive SARS-CoV-2 PCR result was reported, confirming COVID-19. Responsibility for care was formally transferred to the inpatient COVID-19 team, and initial management appropriately prioritized acute COVID-19 care in accordance with mandatory public health isolation requirements and the dominant clinical focus on the pandemic virus at that time.

Importantly, the evolution of antimicrobial management on Day 6 illustrates a structured, multi-stage clinical reasoning process rather than premature diagnostic closure. Upon urgent admission at 18 : 30, care was transferred from the outpatient physician to the inpatient team. In accordance with institutional isolation protocols, the inpatient team initially prioritized acute COVID-19 management, leading to the temporary discontinuation of CAM. However, following initial stabilization, the inpatient team conducted a thorough re-evaluation of the patient’s electronic medical record during the team discussion at 20 : 30. By integrating the outpatient JRS criteria (6/6) and the elevated *M. pneumoniae* PA titre (1 : 640) with the confirmed viral diagnosis, the team recognized that possible *M. pneumoniae* co-detection could not be ruled out. Consequently, intravenous LVFX was initiated to ensure appropriate coverage for potential atypical bacterial co-pathogens.

This sequential decision-making process highlights how team discussions and formal chart re-evaluations during care transitions serve as vital safeguards against cognitive biases – such as anchoring or diagnostic momentum – especially when clinical focus is heavily swayed by an overarching public health crisis.

These contextual factors alone could account for the diagnostic shift without requiring cognitive explanations. Similar cognitive patterns have been described in early-pandemic diagnostic analyses [3–5]. Retrospective interpretation suggests that the sequence in which diagnostic information became available, combined with overlapping radiological findings and limited diagnostic resources, likely contributed to the complexity of clinical reasoning. Importantly, clinicians continued antimicrobial therapy after hospitalization, indicating that alternative diagnoses were not prematurely dismissed.

Radiological overlap between *M. pneumoniae* pneumonia and early COVID-19 has been well described. Ground-glass opacities are common in both conditions, and imaging alone cannot reliably distinguish between them. Early-pandemic reports also demonstrated that detection of another respiratory pathogen does not exclude COVID-19, with co-detection rates varying widely across settings [6–10]. These findings underscore the importance of maintaining a broad differential diagnosis, particularly when diagnostic resources are limited. Although this case occurred in 2020, it offers relevant lessons for contemporary practice. Atypical bacterial infections, including *M. pneumoniae*, have resurged following reduced circulation during the pandemic [11]. These trends highlight the continued importance of maintaining a broad differential diagnosis when evaluating respiratory illness [12].

## Limitations

This report has several limitations. First, it describes a single retrospective case from the early COVID-19 pandemic, and diagnostic pathways at that time differed substantially from current practice. Second, *M. pneumoniae* PCR and multiplex respiratory PCR were not available at our institution, and the diagnosis of *M. pneumoniae* infection relied solely on serology. A single PA titre cannot confirm acute infection, and past exposure or persistent antibody responses cannot be excluded. Third, cognitive processes such as anchoring or premature closure cannot be directly inferred from retrospective documentation; any discussion of cognitive bias represents interpretation rather than definitive evidence. Finally, the temporal relationship between *M. pneumoniae* and SARS-CoV-2 acquisition cannot be determined from available data.

These limitations highlight the need for cautious interpretation and underscore the diagnostic constraints present during the early pandemic.

## Conclusion

This case illustrates the diagnostic complexity encountered during the early COVID-19 pandemic, when limited diagnostic resources, delayed test availability and overlapping radiological findings complicated clinical reasoning. Although *M. pneumoniae* serology and SARS-CoV-2 PCR were both positive early in the clinical course, the evidence is insufficient to confirm acute co-detection. The diagnostic shift observed on Day 6 may have been influenced by the sequence of result availability and early-pandemic diagnostic priorities, although cognitive biases cannot be established definitively.

Even in contemporary practice, where multiplex PCR panels and rapid SARS-CoV-2 and influenza diagnostics are widely available, clinicians must remain attentive to the possibility of co-detection and maintain a broad differential diagnosis when evaluating respiratory illness. Diagnostic complexity can still arise from interpretive variability, the sequence in which results become available, and cognitive influences. Careful, balanced clinical reasoning therefore remains essential for optimal patient management.

## Abbreviations

CAM: clarithromycin
COVID-19: Coronavirus disease 2019
JRS: Japanese Respiratory Society
LVFX: levofloxacin
PA: particle agglutination
SARS-CoV-2: severe acute respiratory syndrome coronavirus 2
